# More Efficient Prussian Blue Nanoparticles for an Improved Caesium Decontamination from Aqueous Solutions and Biological Fluids

**DOI:** 10.3390/molecules25153447

**Published:** 2020-07-29

**Authors:** Fabio Carniato, Giorgio Gatti, Chiara Vittoni, Andrey M. Katsev, Matteo Guidotti, Claudio Evangelisti, Chiara Bisio

**Affiliations:** 1 Dipartimento di Scienze e Innovazione Tecnologica and “Centro interdisciplinare Nano-SiSTeMI”, Università del Piemonte Orientale, via T. Michel 11, 15121 Alessandria, Italy; fabio.carniato@uniupo.it (F.C.); giorgio.gatti@uniupo.it (G.G.); chiara.vittoni@uniupo.it (C.V.); 2 Medical Academy, V.I. Vernadsky Crimean Federal University, 295051 Simferopol, Ukraine; katsev@mail.ru; 3 CNR-SCITEC Istituto di Scienze e Tecnologie Chimiche “Giulio Natta”, via C. Golgi 19, 20133 Milano, Italy; 4 CNR-ICCOM Istituto di Chimica dei Composti Organo Metallici, via G. Moruzzi 1, 56124 Pisa, Italy; claudio.evangelisti@cnr.it

**Keywords:** biotoxicity, internal decontamination, nanoparticles, Prussian blue, ^137^Cs removal

## Abstract

Any release of radioactive cesium-137, due to unintentional accidents in nuclear plants, represents a dangerous threat for human health and the environment. Prussian blue has been widely studied and used as an antidote for humans exposed to acute internal contamination by Cs-137, due to its ability to act as a selective adsorption agent and to its negligible toxicity. In the present work, the synthesis protocol has been revisited avoiding the use of organic solvents to obtain Prussian blue nanoparticles with morphological and textural properties, which positively influence its Cs^+^ binding capacity compared to a commercially available Prussian blue sample. The reduction of the particle size and the increase in the specific surface area and pore volume values compared to the commercial Prussian blue reference led to a more rapid uptake of caesium in simulated enteric fluid solution (+35% after 1 h of contact). Then, after 24 h of contact, both solids were able to remove >98% of the initial Cs^+^ content. The Prussian blue nanoparticles showed a weak inhibition of the bacterial luminescence in the aqueous phase and no chronic detrimental toxic effects.

## 1. Introduction

The synthesis of Prussian blue (PB) has been known since the early 18th century [1,2]. Used for a long time as a painter’s pigment and dye for textiles, since the early 1960s it has been studied and subsequently widely used as an antidote for internal contamination due to radioactive caesium-137 or to toxic thallium salts [3]. Caesium-137 undergoes radioactive decay with the emission of high-energy beta and secondary gamma radiations with a half-life of ca. 30.17 years. The ingestion or inhalation of particles containing this radioisotope can cause severe health effects, especially in the case of internal contamination occurring in the proximity of vital organs [4].

PB is a member of hexacyanometallate compounds, which can exist in aqueous media as ‘soluble Prussian blue’ (KFe^III^{Fe^II^(CN)_6_}·nH_2_O) and as ‘insoluble Prussian blue’ (Fe_4_^III^{Fe^II^(CN)_6_}_3_·nH_2_O) [5,6]. Actually, both species are not soluble in water, but these terms refer to the capacity of PB to be dispersed as a colloidal suspension [7]. The lattice structure of PB is a three-dimensional polymeric structure with a face-centered cubic cells, featuring alternating Fe(III) and Fe(II) ions connected by bridging cyanide ligands [8,9]. Since the diameter of the lattice channels is 3.2 Å wide, the PB structure is capable to accommodate efficiently water molecules and heavy metal cations [10]. It therefore acts as a selective sequestering agent of cationic radioisotopes of caesium or (non-radioactive) thallium species [11,12] and, due to its reduced toxicity, it can be applied under in vivo conditions, in humans subjected to acute exposure [13,14]. After oral ingestion, PB is indeed able to decrease the biological half-life of radioactive Cs, by clearing the fraction of the hazardous metal species present into the gut, enhancing their excretion from the gastrointestinal tract, preventing their reabsorption and mitigating their diffusion within the body. The exact mechanism of Cs binding by PB is still debated. However, it is typically attributed to a cooperative effect of physical adsorption, ion trapping within the lattice channels and ion exchange of pristine potassium and hydronium cations present in its structure for caesium [3,15,16].

More recently, due to the discovery of some interesting specific properties, PB has been attracting again great attention across the international scientific community becoming, together with its analogues, a versatile material for a wide range of applications, such as in sensors [17], catalysts [18,19], batteries [20,21], electrochromic devices [22], and biomedical systems [23,24].

However, the original use of PB as an antidote for the internal decontamination agent from radioactive isotopes is now living a new springtime. Actually, the growing number of nuclear power plants at the worldwide level (441 operating in the first half of 2020 and 54 under construction) [25] gives rise to an ever-increasing risk of major accidents linked to the unintentional release of large amounts of contaminating radioisotopes, as it was the case in Chernobyl in 1986 and Fukushima in 2011. Analogously, the potential use for criminal purposes of radioactive sources or explosive radiological dispersal devices, RDD, is felt as a growing point of concern too [26,27]. The uncontrolled dispersion of radioactive contamination would lead to dramatic long-term effects in terms of radiation-related pathologies and psychological problems. A countermeasure to such a threat thus needs an effective, cheap, and widely available decontamination method.

Recent studies are “re-discovering” the effectiveness of Prussian blue as a sequestering agent for both environmental and medical applications, i.e., for the decontamination of liquid effluents from damaged nuclear power plants or in the case of radioactive isotope leaking as well as for the treatment of patients suffering from acute ^137^Cs internal contamination. In the first case, the research focused on the immobilization of PB over different matrices, in order to make its recovery easier. For example, structured and/or porous materials, such as graphene oxide [28], polymers [29,30], carbohydrates [12,31], mesoporous silicas [32], and clays [33], were used as supports and the relative composite materials showed a high caesium adsorption capacity associated with high selectivity. However, some of these complex systems may require a careful control of their preparation method, are time consuming and this often leads to high production costs and limited applicability of the materials in practical cases. However, many studies about Prussian blue deposited on nanoparticles have appeared recently in the literature both for environmental and biomedical practical applications [34,35,36,37]. Prussian blue is also particularly suitable for medical applications, as its toxicity on humans is very modest. A low level of its toxicity was confirmed by experiments on living organisms of different levels such as laboratory rodents, dogs, ruminants and humans, with lethal dose values attaining >8000 mg per kilogram of body weight (in mice, rats and rabbits) [38,39].

In the present work, in order to reduce the environmental impact and to improve its suitability of biomedical applications, Prussian blue preparation in the form of nanoparticles has been modified avoiding the use of organic solvents, often required to obtain highly pure products [40]. In particular, the purification step has been optimized following the green chemistry principles, avoiding the use of acetone or other environmentally unfriendly organic solvents, without compromising the quality and the purity of the material. The improvement of the synthetic process with a clean, effective, and cheap method, avoiding the use of organic solvents is of particular relevance.

The physicochemical properties of the obtained Prussian blue nanoparticles have been studied by using a multi-technique approach, focusing on those that can influence the caesium sequestration process (i.e., morphological and textural properties) and hence positively affecting the overall process of radioactive metal removal from the living organism. A constant attention was, in parallel, paid to the assessment of potential acute or chronic biotoxicity effects of such optimized forms of PB.

## 2. Results and Discussion

### 2.1. Physicochemical Characterization of Prussian Blue Samples

Prussian blue particles were synthesized by modifying the coprecipitation methods currently present in the literature in order to reduce the environmental impact of the overall process. In particular, the purification step has been optimized avoiding the use of acetone or other environmentally unfriendly organic solvents that have often been employed in the recent literature [41,42,43,44] in order to obtain high-performance materials, suitable for biological and/or medica applications, without compromising the quality and the purity of the material.

The chemical composition of PB samples (a benchmark commercial sample, PB_Com, and the one prepared in the present work, PB-Syn) was determined by elemental C, H, N analysis and the content in carbon, hydrogen, and nitrogen is reported in Table S1 (Supplementary materials).

The carbon to nitrogen ratio of 0.865 and 0.926, for PB_Com and PB_Syn, respectively, were close to the theoretical ratio of 0.858 computed for the formula of the anhydrous Fe_4_[Fe(CN)_6_]_3_ sample. However, taking into account the hydrogen content too, the presence of ca. 20 molecules of water can be foreseen. Indeed, the theoretical values for Fe_4_[Fe(CN)_6_]_3_∙20H_2_O with: C = 17.8%, H = 3.3% and N = 20.7%, were fully in agreement with the data recorded on both samples.

The structural properties of Prussian blue samples were investigated by X-ray diffraction analysis (Figure S1 in Supplementary materials).

The XRD pattern of the reference commercial PB sample and the sample prepared in this work, named PB_Com (Figure S1, curve a) and PB_Syn (Figure S1, curve b), show reflections at 17.4, 24.6, 35.2, 39.2, 43.7, 51.4, 54.5, and 57.5° 2θ, corresponding to the planes (200), (220), (400), (420), (422), (440), (600), and (620), respectively. These peaks can be indexed to iron(III) hexacyanoferrate(II) lattice with a face-centered cubic structure (JCPDS card No 52-1097). It can also be noted that for the PB_Syn sample the reflections appeared wider compared to those obtained in the case of PB_Com material. This behavior gives a first indication on the reduced particle size of the PB_Syn solid.

**Figure 1:**
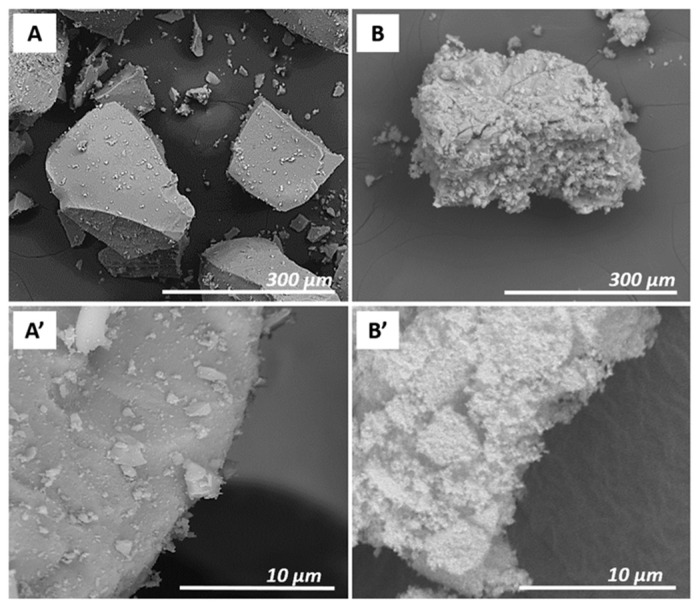
SEM images of PB_Com (**A**,**A’**) and PB_Syn (**B**,**B’**) at 500× and 10,000× magnifications.

The morphology of Prussian blue samples was preliminarily studied by using an SEM analysis. SEM images of PB_Com (A, A’) and PB_Syn (B, B’) samples are shown in Figure 1.

The PB_Com sample (Figure 1, Frame A) was constituted by square-shaped aggregates with micrometric size (about 200–600 μm). These aggregates were formed by particles with a smaller dimension, better visible in the Frame A’. The PB_Syn sample (Figure 1, Frame B), on the contrary, was characterized by aggregates of less regular shape with a micrometric size (50–300 μm). These aggregates were made of spherical particles with a smaller size compared to the commercial sample (100–400 nm; Frame B’). However, a correct estimation of the size of the particles, which compose of these aggregates, is difficult to be appreciated by SEM micrographs. For this reason, additional details on the morphology, particles size and elemental composition at the nanometric scale of Prussian blue samples were obtained by transmission electron microscopy experiments.

**Figure 2:**
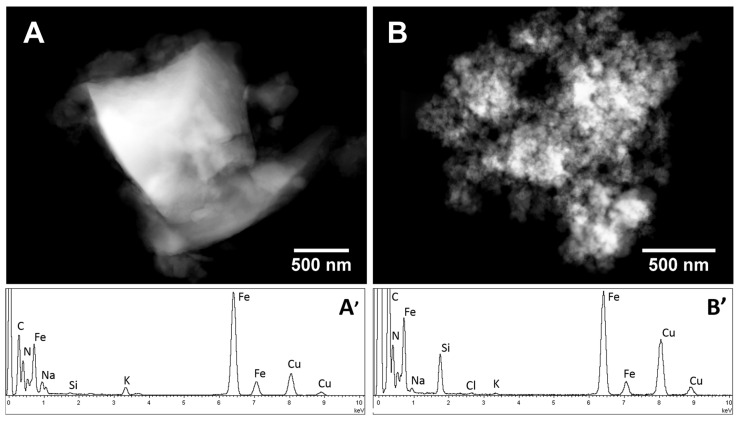
Representative HAADF-STEM micrograph and corresponding EDX spectrum of the PB_Com (**A**,**A’**, respectively) and PB_Syn (**B**,**B’**, respectively) sample.

**Figure 3:**
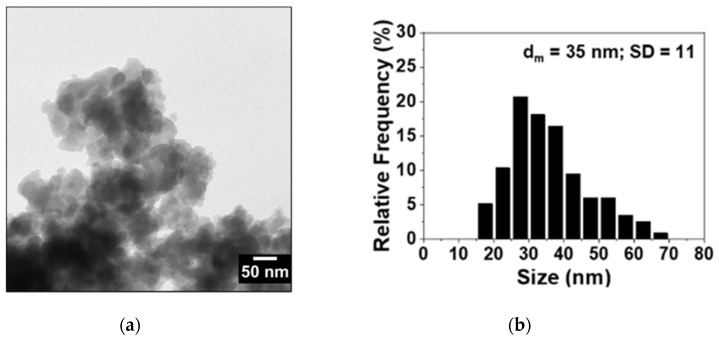
Representative TEM micrograph (**a**) and histogram of particles size distribution (**b**) of the PB_Syn sample.

HAADF-STEM (High Angle Annular Dark Field- Scanning Transmission Electron Microscopy) analysis of the PB_Com sample (Figure 2A) revealed the presence of rather large crystals (1–5 µm) having a compact and non-porous structure. On the other hand, the PB_Syn (Figure 2B) shows aggregates similar in size but composed of much smaller crystals. TEM analysis carried out at a higher magnification confirms the presence of nanocrystals ranging from 20 to 70 nm in size with a mean diameter of 35 nm (see Figure 3).

All these morphological data revealed an important ability of both PB particles to generate large aggregates. This was also evident when both samples were suspended in pure water. Dynamic light scattering (DLS) data show the presence of aggregates with a hydrodynamic diameter from 200 and 500 nm (Figure S2 in Supplementary materials).

The EDX analysis (Figure 2A’,B’, respectively) shows for both samples the signals of iron, nitrogen, and carbon, as expected from the structure of the Prussian blue salt. Copper and carbon signals were due to the TEM support grid. By comparing the EDX spectra of the two samples, it is worth noting that the amount of potassium related to the amount of iron (K/Fe molar ratio 0.08) was larger in the PB_Com sample than in PB-Syn (K/Fe molar ratio 0.01). Considering the almost total absence of chlorine signals in PB_Com sample grains, the presence of the KCl could be excluded. Therefore, the observed potassium signal could be ascribed to the presence of hexacyanoferrate mixed salts, as byproducts deriving from the PB synthesis, in the benchmark PB_Com solid. On the other hand, in the PB_Syn sample, an equimolar amount of K and Cl was detected (less than 1 mol% with respect to Fe) suggesting the presence of a very small amount of KCl. Moreover, in the commercial sample, potassium and sodium chloride salts segregated out of the PB grains were observed (see Figure S3 in Supplementary materials). Conversely, no segregated chlorides salts were observed in PB_Syn.

Such a remarkable difference in the particle size of the two PB samples was evident in the profiles of thermogravimetric analysis conducted in ultrapure air flow with a 5 °C·min^−1^ heating rate (Figure S4, Frame A). Both solids displayed a gradual weight loss up to 160 °C, attributable to weakly bound water molecules. Indeed, considering the chemical formula Fe_4_[Fe(CN)_6_]_3_·20H_2_O, as suggested by the C, H, N elemental analysis (vide supra), interstitial water molecules should account for ca. 29.5% of the overall weight. Indeed, in the weight loss profiles, the first plateaus corresponded roughly to 23% (ca. 16 water molecules for formula unit) and 26% (corresponding to ca. 18 water molecules for formula unit), for PB_Com and PB_Syn, respectively, which were in line with the hypothesis of water content for these solids. Then, the thermal degradation of the inorganic structure takes place at ca. 230 °C for PB_Syn and around 325 °C for PB_Com samples. The shift towards lower temperatures of the degradation step for PB_Syn with respect to the commercial sample is further evidence of the different particles size of the two compounds. The same trend was observed by performing TGA analysis in different experimental conditions, under argon flow and with a heating rate of 10 °C·min^−1^ (Figure S4, Frame B in Supplementary materials).

**Figure 4:**
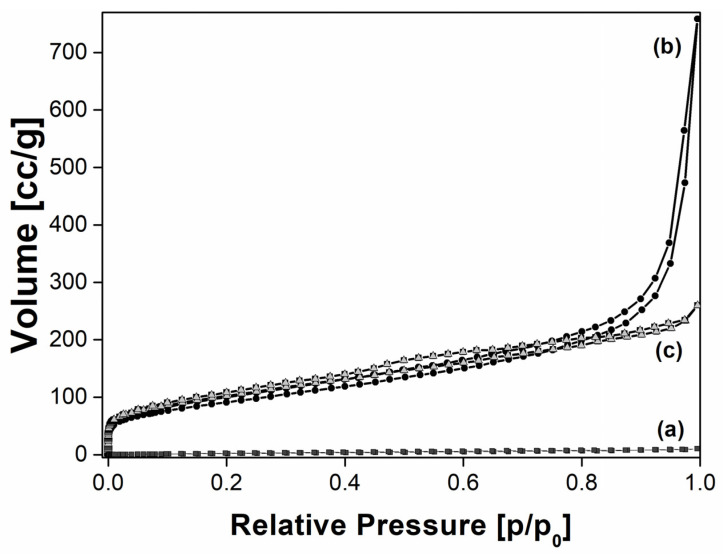
N_2_ Physisorption at −196 °C at relative pressures (p/p_0_) from 1 × 10^−7^ to 1 of: PB_Com (■, **a**), PB_Syn (●, **b**), and PB_Acid (∆, **c**) samples.

Finally, the textural properties of the Prussian blue samples have been studied through N_2_ physisorption analysis. The obtained isotherms are reported in Figure 4.

The PB_Com sample was characterized by an exceedingly low adsorption of N_2_ (Figure 4, curve a). Oppositely, the adsorption isotherm of the PB_Syn sample (Figure 4, curve b) belonged to Type II (IUPAC classification), typical of non-porous solids, with a thin hysteresis loop of the H1 type, due to the aggregation of the PB particles that generate a non-structural porosity, in a wide range between 0.1 and 1 p/p_0_. The Brunauer–Emmett–Teller (BET) model was used to estimate the specific surface area of the materials that was determined to be 10 m^2^·g^−1^ and 330 m^2^·g^−1^, respectively for PB_Com and PB_Syn samples. The specific pore volume values, determined by using the NLDFT method, were found to be 0.014 cm^3^·g^−1^ and 0.794 cm^3^·g^−1^ for PB_Com and PB_Syn samples, respectively.

Such a significant difference in Specific Surface Area (SSA) and specific pores volume values for PB_Syn sample compared to PB_Com is attributed to the different size of the crystals.

### 2.2. Simulation of Caesium Removal from Enteric Fluid

**Figure 5:**
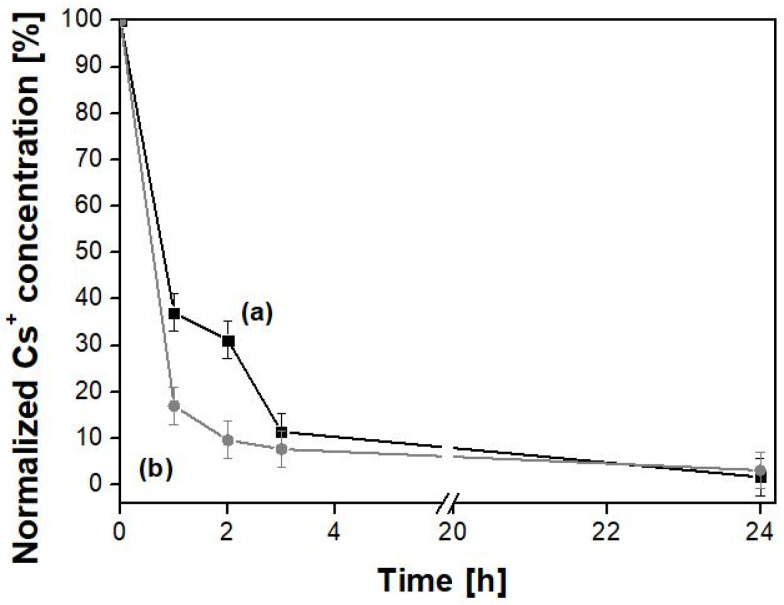
Normalized Cs^+^ concentration in enteric fluid after contact with PB_Com (■, **a**) and PB_Syn (●, **b**) samples for 1, 2, 3, and 24 h. Test conditions: total volume = 10 mL; initial ^133^Cs concentration = 500 mg/L; PB mass = 20 mg; room temperature. Standard error achieved from the repetition of 3 measurements. A break from 5 h to 20 h is reported in the graph.

The ability of PB_Com and PB_Syn samples to remove (non-radioactive) Cs^+^ ions in the case of acute intoxication was evaluated by measuring the residual dissolved Cs^+^ concentration after contacting the PB samples with a simulated enteric fluid solution (Figure 5).

Both Prussian blue samples removed, after 24 h, more than 98% of the caesium previously present in the enteric fluid solution. Notwithstanding their different origin and textural characteristics, the two samples showed a similar behavior for long contact times. However, in order to effectively mitigate the detrimental effects of radio-caesium on living organisms and to shorten its biological half-life by influencing the equilibrium of the natural decorporation process, it is crucial to attain a rapid removal of Cs species in the first hours of transit through the gastro-enteric tract [3]. Indeed, the longer the residence of the emitting species within the body, the larger the damage to the tissues, especially due to the beta emission properties.

With this aspect in mind, the PB_Syn sample (Figure 5, curve b) showed an interesting behavior: after 1 h of contact, this sample seized about 85% of the caesium present in the solution (corresponding to 212.5 mg/g), whereas the PB_Com sample (Figure 5, curve a) seized only 63% of it after the same time (corresponding to 157.5 mg/g). The higher caesium removal capability of the former than the latter sample can be ascribed to two linked factors: (i) a smaller particle size, and therefore (ii) a higher specific surface area of the tailored sample with respect to the commercial material.

The data were also quantitatively analyzed by using a parabolic diffusion model describing diffusion-controlled events (Figure S5 in Supplementary materials) [45] by the following equation:

(1 − M_t_/M_0_)/t = −kt^−0.5^ + m (1)

where M_0_ and M_t_ are the amount of Cs^+^ ions in Prussian blue samples at time 0 and t, respectively, k is the rate constant, and m is a constant of which the chemical significance is not clearly resolved [46,47]. The k values obtained for PB_Com and PB_Syn samples were 0.63 and 0.82 s^−1^, respectively.

### 2.3. Effect of Gastric Fluid

Since the ingested PB solid is expected to pass through the stomach before reaching the gut, where it comes into contact with the radioactive caesium acting as a clearing agent, the effect of the harsh conditions found in the gastric fluid on the PB samples was preliminarily investigated. In particular, the variation in the physicochemical properties and the ^133^Cs seizure capacities of Prussian blue were evaluated after the contact with a gastric fluid simulant solution.

The PB_Acid sample was characterized and compared with the pristine PB_Syn sample with the aim of evaluating any possible modification of the structural, morphological, and textural properties after contact and interaction with the gastric fluid simulant.

The XRD patterns of PB_Syn (a) and PB_Acid (c) samples are reported in Figure S1. The X-ray diffraction analysis showed that the PB_Acid sample (Figure S1, curve c) shows reflections in the same positions as the pristine sample, which did not undergo the acid treatment (PB_Syn, Figure S6, curve b), indicating that the structure was not modified after the contact with the gastric fluid. However, in the diffraction pattern of the PB_Acid sample, a slight decrease in intensity with respect to those of the PB_Syn sample was visible. Such a decrease in reflection intensity suggests a slight loss of the crystalline structure.

**Figure 6:**
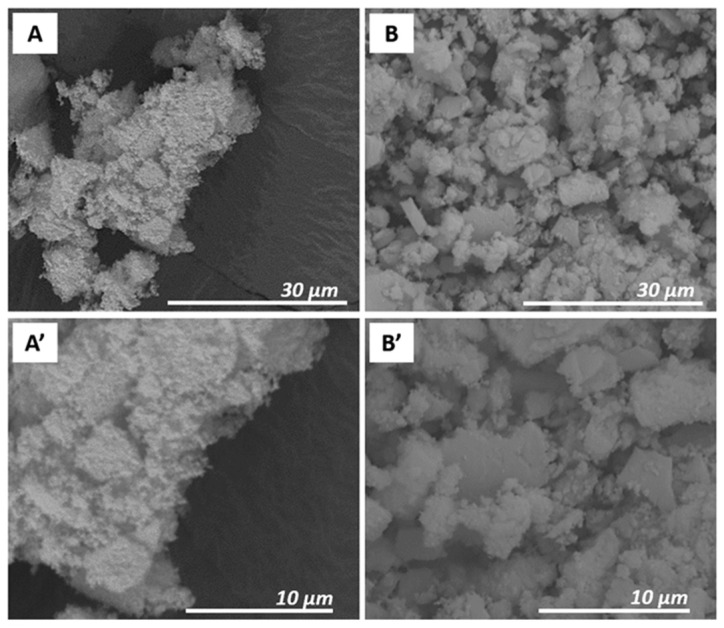
SEM images of PB_Syn (**A**,**A’**) and PB_Acid (**B**,**B’**) at 5000× and 10,000× magnifications.

The effect of the gastric fluid on the morphology of the Prussian blue sample was evaluated by using SEM analysis. The micrographic analysis before (Frames A, A’) and after the acid treatment (Frames B, B’) is reported in Figure 6.

The interaction with the gastric fluid modified the morphology of the aggregates and, in particular, their size. In fact, as can be seen in Figure 6, before the acid treatment the PB sample (Frame A) consisted of aggregates with dimension between 50 and 200 μm, while after acid treatment (Frame B) the dimension of the aggregates decreased to 5–20 μm. This variation of the morphology of the PB_Syn sample is likely due to the strong acidity of the gastric fluid simulant. For both samples, however, the aggregates were composed of smaller particles, with a size of about few hundred nanometers (frames A’ and B’).

The change in textural characteristics of PB_Syn after the contact with the gastric fluid simulant was evident by means of N_2_ adsorption analysis at −196 °C (Figure 4). First, a variation of the shape of the isotherm could be observed. Indeed, the isotherm of the PB_Acid sample (Figure 4, curve c) presents a more marked hysteresis loop than the PB_Syn sample (Figure 4, curve b), thus indicating an increase in the contribution of aggregation porosity.

This effect was due to the reduction of size of the aggregates after the acidic treatment, as previously observed by the SEM analysis too.

The BET specific surface area value of the PB_Acid sample was 370 m^2^·g^−1^, slightly higher than the one of the non-treated sample, i.e., 330 m^2^ g^−1^. This variation in SSA is probably due to the formation of defects in the crystalline structure due to the strong acidity of the gastric fluid simulant. The total pore volume, determined by using the Density Functional Theory (DFT) model, remains unchanged (pore volume values: 0.803 and 0.794 cm^3^ g^−1^, for the PB_Acid and PB_Syn samples, respectively).

**Figure 7:**
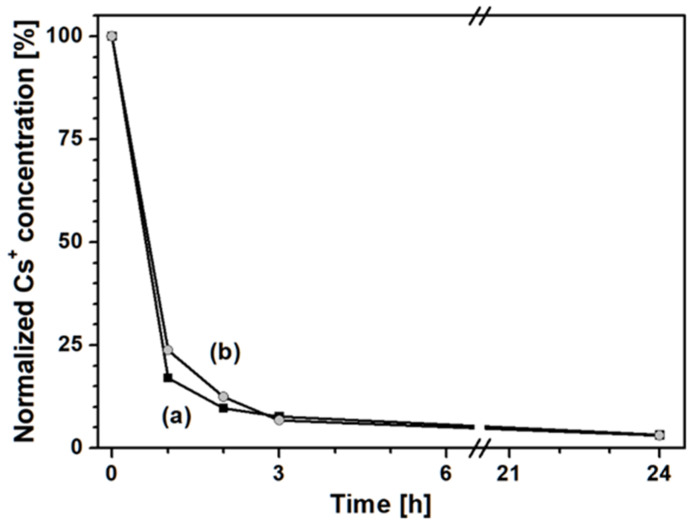
Normalized Cs^+^ concentration in gastric fluid after contact with PB_Syn (■, **a**) and PB_Acid (●, **b**) samples. A break from 6 h to 21 h is reported in the graph.

Finally, the ability of the PB_Acid sample in removing Cs^+^ species from a contaminated enteric fluid after contact with gastric fluid was evaluated (Figure 7).

PB after 24 h of contact with a gastric fluid simulant (Figure 7, curve b) was able to remove the same amount of Cs^+^ (i.e., 98%) as the non-treated Prussian blue sample (Figure 7, curve a). Only a slight decrease in the caesium capture ability was observed in the first 3 h of test. However, the changes in the physicochemical properties observed over the PB_Acid sample did not lead to significant changes in terms of the ability of PB to remove Cs ions from a simulated enteric fluid, after a simulated transit through the gastric tract. Such results suggest a good stability of the optimized PB material against attacks under strongly acid conditions.

### 2.4. Toxicological Evaluation: Biotests

**Figure 8:**
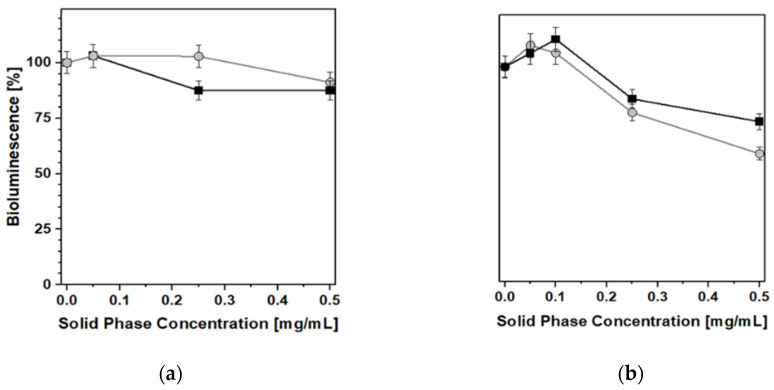
Acute effect of ● PB_Syn and ■ PB_Com samples on *Photobacterium leiognathi Sh1* (**a**) and *Aliivibrio fischeri F1* (**b**) bioluminescence. 10 min, T = 25 °C.

Both PB_Syn and PB_Com samples were relatively easily suspended in the aqueous solutions needed for the biotests, the former being more hydrophilic and more akin to form metastable aqueous suspensions. Nevertheless, despite the notable differences in physicochemical properties shown above, the effect of the two samples on bacterial luminescence was quite similar. In acute toxicity tests (Figure 8), both PB samples exhibited only a weak inhibition of bacterial luminescence and growth, at concentrations higher than 0.25 mg/mL, at 25 °C, after 10 min, for *Photobacterium leiognathi Sh1* (Frame a) and *Aliivibrio fischeri F1* (Frame b). In these experiments, the behavior of both PB solids was almost comparable and no major differences between the two materials were observed.

**Figure 9:**
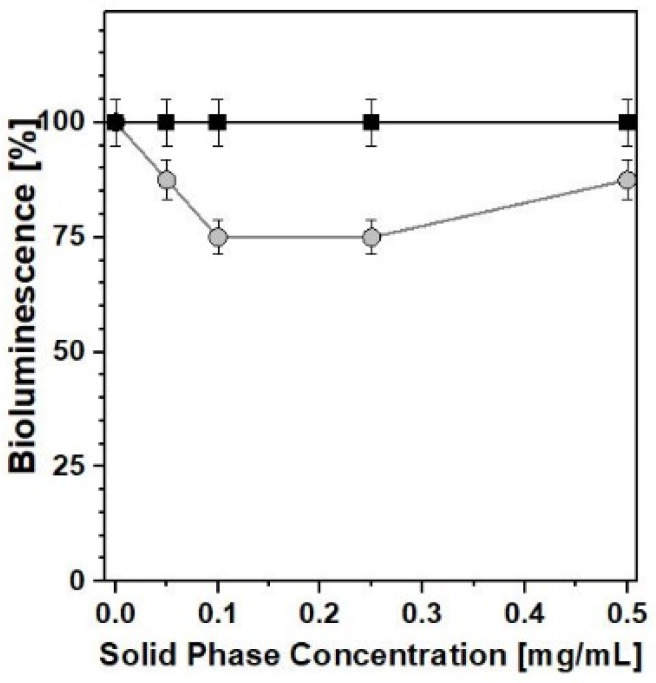
Chronic effect of ● PB_Syn and ■ PB_Com samples on *A. fischeri F1* bioluminescence. 24 h, T = 25 °C.

Conversely, with regard to the chronic toxicity test (Figure 9), some minor inhibition of luminescence (a decrease of ca. 25%) was recorded, after 24 h, in the presence of PB_Syn, whereas no negative effects at all were detected when the reference PB_Com solid was used as a caesium removal agent. This was the only case in which the relevant differences in textural and morphological characteristics gave rise to a clearly detectable difference in biotoxicity behavior.

**Figure 10:**
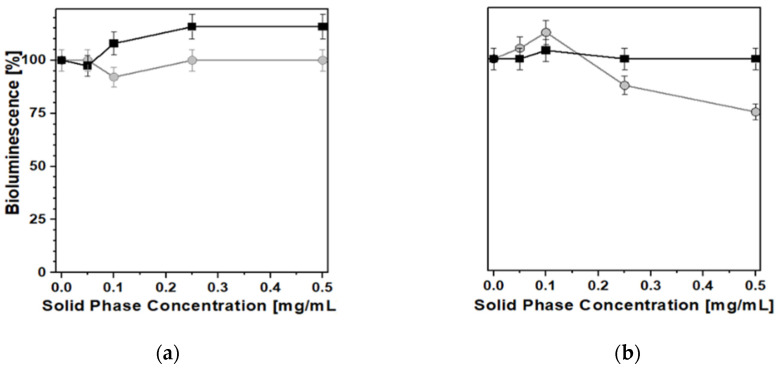
Acute (2 h; **a**) and chronic (24 h; **b**) effects of ● PB_Syn and ■ PB_Com samples on *E. coli* (pXen7) bioluminescence. T = 37 °C.

A similar set of results was obtained by performing the biotests for general toxicity with the help of recombinant test-bacteria *E. coli* (pXen7), characterized by a constitutive bioluminescence that is similar to the performance of marine bioluminescent test-bacteria, but allowed us to study the PB samples under conditions closer to the physiological ones. In this case, all biotests were performed at 37 °C, taking into account the optimum growth temperature for *E. coli*. No significant changes in bioluminescence were observed after 2 h of incubation (Figure 10a) or in the time range of 18–24 h (Figure 10b).

**Figure 11:**
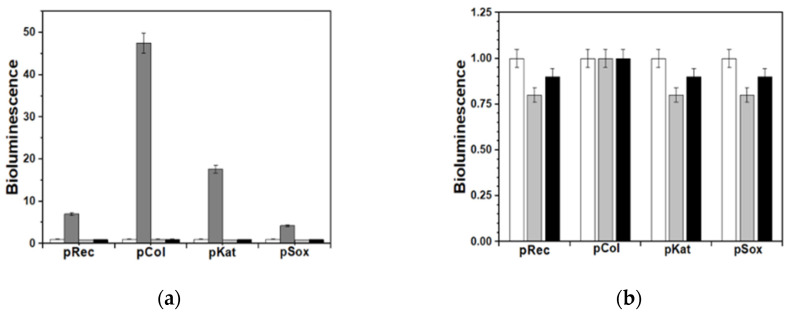
Response of bioluminescent bacterial bioreporters to ■ PB_Syn and ■ PB_Com samples (□ Blank and ■ Control). Graph (**a**): Effects of PB samples in comparison with bioluminescence induction by dioxidine (2.25 × 10^−5^ M) and aqueous H_2_O_2_ (4.45 × 10^−4^ M). Graph (**b**): Bioluminescence of the bioreporter test-bacteria in the presence of PB samples, in comparison with a blank.

Further studies about some specific kinds of toxicity were carried out using four bioreporting strains (see Experimental section). The results showed the absence of capability for either PB sample to cause damages to nucleic acids as well as to induce oxidative stress (Figure 11a,b). These data are fully consistent with the broad previous literature reporting on the extremely low risks connected to the use of PB as a decorporation agent for caesium and/or thallium internal contamination [38,39].

## 3. Materials and Methods

### 3.1. Materials Preparation and Supplying

Commercial Prussian blue (PB_Com) was obtained from Heyl pharmaceutical company (Berlin, Germany). The drug is marketed under the designation “Antidotum Thallii-Heyl”, in the form of hard capsules containing 500 mg of active substance. The capsules contained 68% of iron(III) hexacyanoferrate(II) (Fe_4_[Fe(CN)_6_]_3_) and as other co-ingredients microcrystalline cellulose, gelatine, indigo carmine (E132), sodium dodecyl sulfate, and water for injection.

The synthetic Prussian blue sample (PB_Syn) was prepared as follows: 2.6 g of iron(III) chloride hexahydrate (FeCl_3_∙6H_2_O, MM = 270.3 g·mol^−1^, Sigma Aldrich) and 1.0 g of potassium hexacyanoferrate(II) trihydrate (K_4_[Fe(CN)_6_]·3H_2_O, MM = 422.3 g·mol^−1^, Sigma Aldrich, St. Louis, MO, USA) were separately dissolved in 10 mL of deionized water. Then, the solution of K_4_[Fe(CN)_6_] was added dropwise to the FeCl_3_ solution and immediately the formation of a blue precipitate was observed. The dispersion was left under stirring for 10 min, after which the insoluble form of Prussian blue was filtered and washed with about 500 mL of deionized water. Finally, the obtained sample was dried in an oven at 80 °C for 5 h to remove any traces of water. Following this procedure, about 0.9 g of Fe_4_[Fe(CN)_6_]_3_∙6H_2_O were obtained.

PB_Acid: the sample was prepared by dispersion of 100 mg of PB_Syn in 10 mL of the gastric simulant. The suspension was stirred for 24 h at room temperature and the powder was isolated by two centrifugation cycles (6000 r.p.m. for 10 min). The final solid was dried in an oven for 16 h at 80 °C.

### 3.2. Characterization Techniques

X-ray diffractograms (XRD) were collected on unoriented ground powders using a Thermo ARL ‘XTRA-048 diffractometer (Thermo Fisher Scientific, Waltham, MA, USA) with a CuK_α_ (λ= 1.54 Å) radiation, from 10 to 70 2θ degrees with a step size of 0.02° 2θ and a rate of 1° 2θ·min^−1^.

Scanning electron microscopy (SEM) images were obtained by means of an ESEM Quanta 200 instrument (FEI Company, Hillsboro, OR, USA) in the high vacuum mode. Before the analysis samples were mounted on aluminum stubs by using a conductive carbon tape and gold-coated by using a sputter coater (about 20 Å gold in 20 s) to promote the conductivity.

Dynamic light scattering (DLS) measurements were performed by using a Zetasizer NanoZS (Malvern Instruments; Malvern, UK) operating in a particle size range from 0.6 nm to 6 μm, equipped with a He-Ne laser with λ = 633 nm. Before the analysis the samples were dispersed in ultrapure water (2.5 mg·mL^−1^) and the dispersions were sonicated for 10 min.

Transmission electron microscopy (TEM) and high angular annular dark field scanning transmission electron microscopy (HAADF-STEM) analyses were performed by using a ZEISS LIBRA 200FE microscope (Zeiss, Oberkochen, Germany) equipped with a 200 kV FEG source. Energy-dispersive X-ray spectra (EDS-Oxford INCA Energy TEM 200) were collected along with HAADF-STEM micrographs (Oxford Instruments, Abingdon-on-Thames, UK). The samples were suspended in isopropanol and sonicated, then each suspension was dropped onto a carbon film-coated copper grid (300 mesh) and the solvent was evaporated.

N_2_ physisorption measurements were performed by using a Quantachrome Autosorb iQ2 volumetric system (temperature: −196 °C, relative pressure range from 10^−7^ to 1 p/p_0_). Before the analysis samples were treated in vacuum (residual pressure p_0_ 10^−7^ Torr) at 135 °C for 3 h to remove physisorbed water. Specific surface areas values were determined by using the Brunauer–Emmett–Teller (BET) method, in the residual pressure range from 0.01 to 0.1 p/p_0_. Finally, pore size distributions were obtained by applying the non localized density functional theory (NLDFT) method (N_2_ silica kernel based on a cylindrical pore model).

Carbon, hydrogen, and nitrogen (C, H, and N) elemental analyses were performed on a PerkinElmer instrument CHN 2400 Series II (PerkinElmer, Milan, Italy), equipped with a transistor-grade extra-pure oxygen gas cylinder (SIAD, <50 ppm residual N_2_ content) and connected to a Cahn C-30 Microbalance. The analysis was performed on an aliquot of ca.1–2 mg.

Thermogravimetric analysis (TGA) of the PB samples was carried out on a Perkin Elmer 7HT instrument (PerkinElmer, Milan, Italy) under different experimental conditions. In particular measurements were conducted both in ultrapure air (gas flow 20 mL·min^−1^), heating the sample (ca. 15 mg) from 50 to 800 °C with a rate of 5 °C·min^−1^ and in ultrapure argon flow (20 mL·min^−1^), heating the sample (ca. 10 mg) from 50 to 850 °C with a rate of 10°C·min^−1^.

The concentration of Cs^+^ remaining in solution after the uptake tests was determined by using ICP-MS (Inductively Coupled Plasma- Mass Spectrometry) Thermo Scientific X5 Series (Waltham, MA, USA).

### 3.3. Preparation of the Simulant Fluids

The enteric fluid was simulated by dissolving in 6.40 g of disodium hydrogen phosphate (Na_2_HPO_4_), 0.60 g of potassium dihydrogen phosphate (KH_2_PO_4_), and 5.85 g of sodium chloride (NaCl) in 1 L of deionized water [47]. To simulate a case of acute (radioactive) ^137^Cs intoxication, 500 mg of non-radioactive caesium chloride (^133^CsCl) were dissolved in the enteric fluid simulant.

The simulant of gastric fluid was prepared by dissolving 2 g of sodium chloride (NaCl) and 7 mL of concentrated hydrochloric acid (HCl, 37%) in 1 L of deionized water (pH of the solution: 1.2) [47]. After, 100 mg of the PB_Syn sample was dispersed in 10 mL of the gastric simulant, stirred for 24 h at room temperature and subsequently recovered by means of two centrifugation cycles (6000 r.p.m. for 10 min). Finally, the sample was dried in an oven for 16 h at 80 °C. The obtained sample will be hereafter named (PB_Acid).

### 3.4. Simulation of Cs Removal

The ability of Prussian blue samples to remove caesium over time was determined in the following way: 20 mg of the Prussian blue sample were dispersed in 10 mL of Cs-containing enteric fluid simulant (Cs concentration: 500 mg/L) and placed under mechanical stirring, at room temperature. This operation was repeated four times by varying the contact time (1, 2, 3, and 24 h). After, the sample was separated from the solution by filtration on a polyethylene filter (0.22 μm) and centrifuged at 7500 rpm for 15 min. The amount of caesium remained in solution was subsequently determined by ICP-MS analysis.

### 3.5. Toxicological Evaluation

Natural marine luminescent bacteria *Aliivibrio fischeri* F1 isolated from the Black Sea and *Photobacterium leiognathi* Sh1 recently isolated from the Sea of Azov [48] were used as test-organisms. Numerous studies have shown that these strains were highly sensitive to toxic factors of various natures [49]. The biotoxicity of PB samples was evaluated on both acute and chronic effects [49,50] at 25 °C and a concentration of solid phase ranged from 0.05 to 0.5 mg/mL.

In addition, five recombinant bioluminescent bacteria, kindly provided by I.V. Manukhov (Federal State unitary Enterprise, GosNII Genetica, Moscow, Russia), were used to study general nonspecific and some specific kinds of toxicities [51,52]. Among them, one strain, i.e., *E. coli* (pXen7), possessed a constitutive bioluminescence, while the other four ones were bacterial bioreporters with bioluminescence induced by external factors. Among them, two strains: *E. coli* (pRecA-lux) and *E. coli* (pColD-lux) were sensitive to nucleic acids damages. In these cases, an aqueous solution of dioxidine (hydroxymethylquinoxalindioxide, 2.25 × 10^−5^ M) was used as a control. The other two bioreporters *E. coli* (pKatG-lux) and *E. coli* (pSoxS-lux) were sensitive to oxidative stress caused by H_2_O_2_ and superoxide radicals, respectively. To induce their bioluminescence aqueous hydrogen peroxide with a concentration of 4.45 × 10^−4^ M was used as a control. Blank samples contained only bioreporter test-bacteria.

Bioluminescence intensity measurements were performed by luminometers BLM 8801 and Lum-100 (DiSoft, Moscow, Russia). In the tests with marine luminescent test-bacteria and *E. coli* (pXen7), the results were represented as a percentage of bioluminescence of the samples in comparison to the blank sample. In the case of bioreporter strains, bioluminescence level was represented in terms of a factor of bioluminescence induction (IF), as the ratio of the bioluminescence intensity in the presence of a blank-level sample.

## 4. Conclusions

In the present work, nanosized PB particles were synthesized avoiding the use of conventionally used organic solvents, such as acetone, in the purification steps. In the PB_Syn sample, ultrafine particles with an enhanced control on their size and a smaller monomodal, average diameter (100–200 nm) were obtained with respect to a reference commercially-available PB sample, PB_Com. Due to the diminution of the average particle size, the PB_Syn sample was characterized by a specific surface area and pore volume values that were significantly higher than the ones of PB_Com.

When the performance of PB samples were evaluated in the removal of (non-radioactive) Cs^+^ ions in a simulated enteric fluid solution, the two solids displayed a practically identical result in terms of the total amount of caesium removed at the end of the 24 h-long test (98% removal). However, the tailored PB sample showed an unexpected more rapid uptake of metal than the commercial reference solid: after the first hour of contact, an enhancement of 35% in caesium removal was observed for PB_Syn compared to the PB_Com sample. Such an increase takes place in the initial critical time window for a more effective decorporation of hazardous radio-caesium and is attributed to the enhanced characteristics shown by the PB_Syn sample, linked to a higher surface area, thus increasing the availability of absorption and/or exchange sites over the particles. Then, the potential biotoxicity of such an enhanced form of PB was tested over responsive bioluminescent bacteria. Weak inhibition was observed only in the case of marine bioluminescent test-bacteria. No chronic effects were detected for both PB samples, at least under the conditions tested here.

From the evaluation of the effect of the contact with a simulated gastric fluid, no noteworthy changes in the structural and morphological properties were observed and a minimal increase in terms of specific surface area was recorded, probably due to the formation of defects in the crystalline structure caused by the strong acidity of the gastric fluid simulant. However, none of these (minimal) modifications led to significant changes in the ability to remove caesium ions, demonstrating the effectiveness, safety, and robustness of the Prussian blue sample in the conditions close to the ones of in vivo use.

## Supplementary Materials

The following are available online, Table S1: Elemental C, H, N content of PB_Com and PB_Syn; Figure S1: XRD patterns of (a) PB_Com, (b) PB_Syn, and (c) PB_Acid; Figure S2: Particle size distribution of PB_Com (a) and PB_Syn (b) obtained by a DLS analysis; Figure S3: HAADF-STEM image of segregated KCl grains in PB_Com sample, Figure S4. Frame A: TGA profile of PB_Com and PB_Syn samples. Ultrapure air flow (20 mL·min^−1^); temperature program from 50 to 800 °C, 5 °C·min^−1^; 15 mg sample. Frame: TGA profile of PB_Com (black) and PB_Syn (red) samples. First derivative profile, DTGA, as dashed curves. Ultrapure argon flow (20 mL·min^−1^); temperature program from 50 to 850 °C, 10 °C·min^−1^; 10 mg sample; Figure S5: Plots of the kinetic parabolic model for the Cs^+^ uptake for PB_Syn (A) and PB_Com (B) samples, respectively.
