# Supplementary material for: More Efficient Prussian Blue Nanoparticles for an Improved Caesium Decontamination from Aqueous Solutions and Biological Fluids
Source: Molecules. 2020 Jul 29;25(15):3447. doi: 10.3390/molecules25153447 (PMC7435413; doi:10.3390/molecules25153447)
Supplement: Supplementary file 1 [file molecules-25-03447-s001.pdf]

More Efficient Prussian Blue Nanoparticles for an Improved Caesium Decontamination from Aqueous Solutions and Biological Fluids

Fabio Carniato [1], Giorgio Gatti [1], Chiara Vittoni [1], Andrey M. Katsev [2], Matteo Guidotti [3,*], Claudio Evangelisti [4] and Chiara Bisio [1,*]

[1] Dipartimento di Scienze e Innovazione Tecnologica and "Centro interdisciplinare Nano-SiSTeMI", Università del Piemonte Orientale, via T. Michel 11, 15121 Alessandria, Italy; fabio.carniato@uniupo.it (F.C.); giorgio.gatti@uniupo.it (G.G.) chiara.vittoni@uniupo.it (C.V.)
[2] Medical Academy, V.I. Vernadsky Crimean Federal University, 295051 Simferopol, Crimea; katsev@mail.ru
[3] CNR-SCITEC Istituto di Scienze e Tecnologie Chimiche "Giulio Natta", via C. Golgi 19, 20133 Milano, Italy
[4] CNR-ICCOM Istituto di Chimica dei Composti Organo Metallici, via G. Moruzzi 1, 56124 Pisa, Italy; claudio.evangelisti@cnr.it
* Correspondence: matteo.guidotti@scitec.cnr.it (M.G.); chiara.bisio@uniupo.it (C.B.)

SUPPORTING INFORMATION

Table S1. Elemental C, H, N content of PB_Com and PB_Syn.

Content (wt.%)	PB_Com	PB_Syn
C	17.57	17.82
H	2.91	3.31

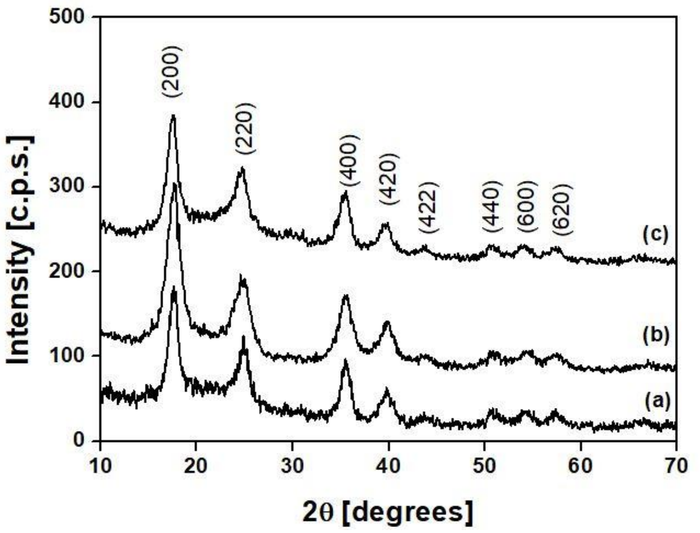

Figure S1. XRD patterns of (**a**) PB_Com (**b**) PB_Syn and (**c**) PB_Acid.

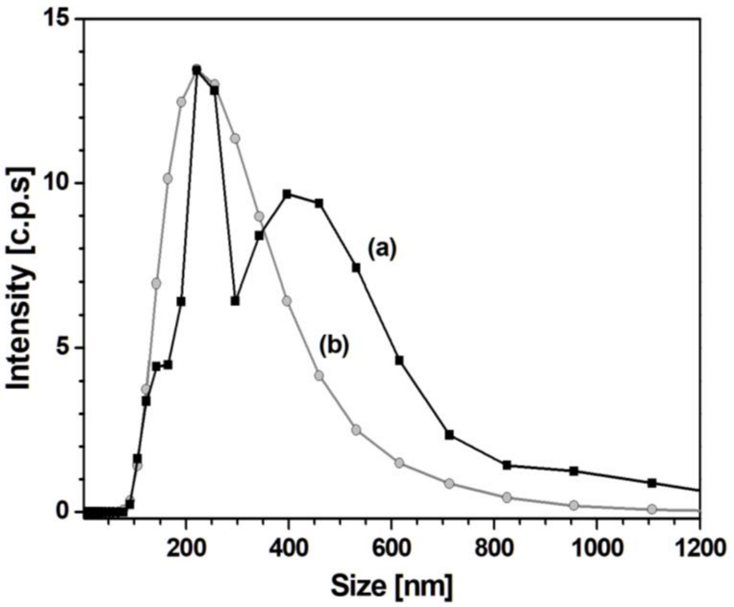

Figure S2. Particle size distribution of PB_Com (**a**) and PB_Syn (**b**) obtained by DLS analysis.

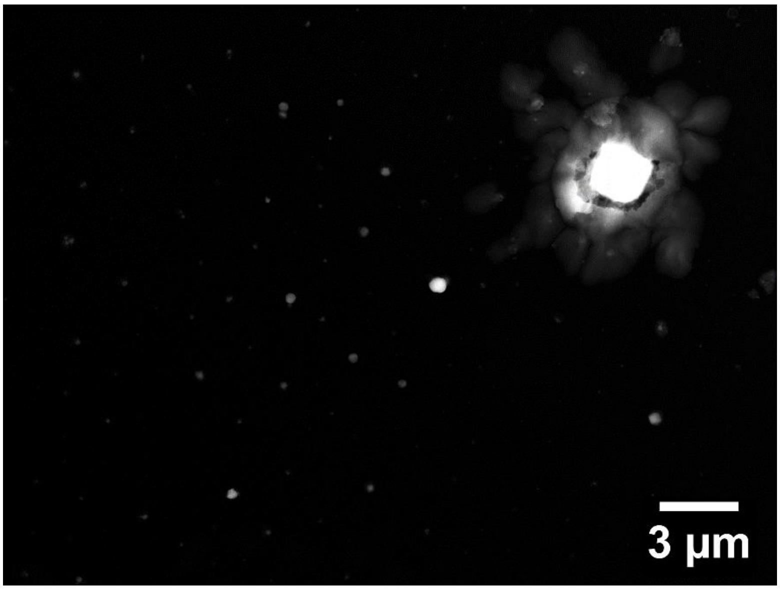

Figure S3. HAADF-STEM image of segregated KCl grains in PB_Com sample.

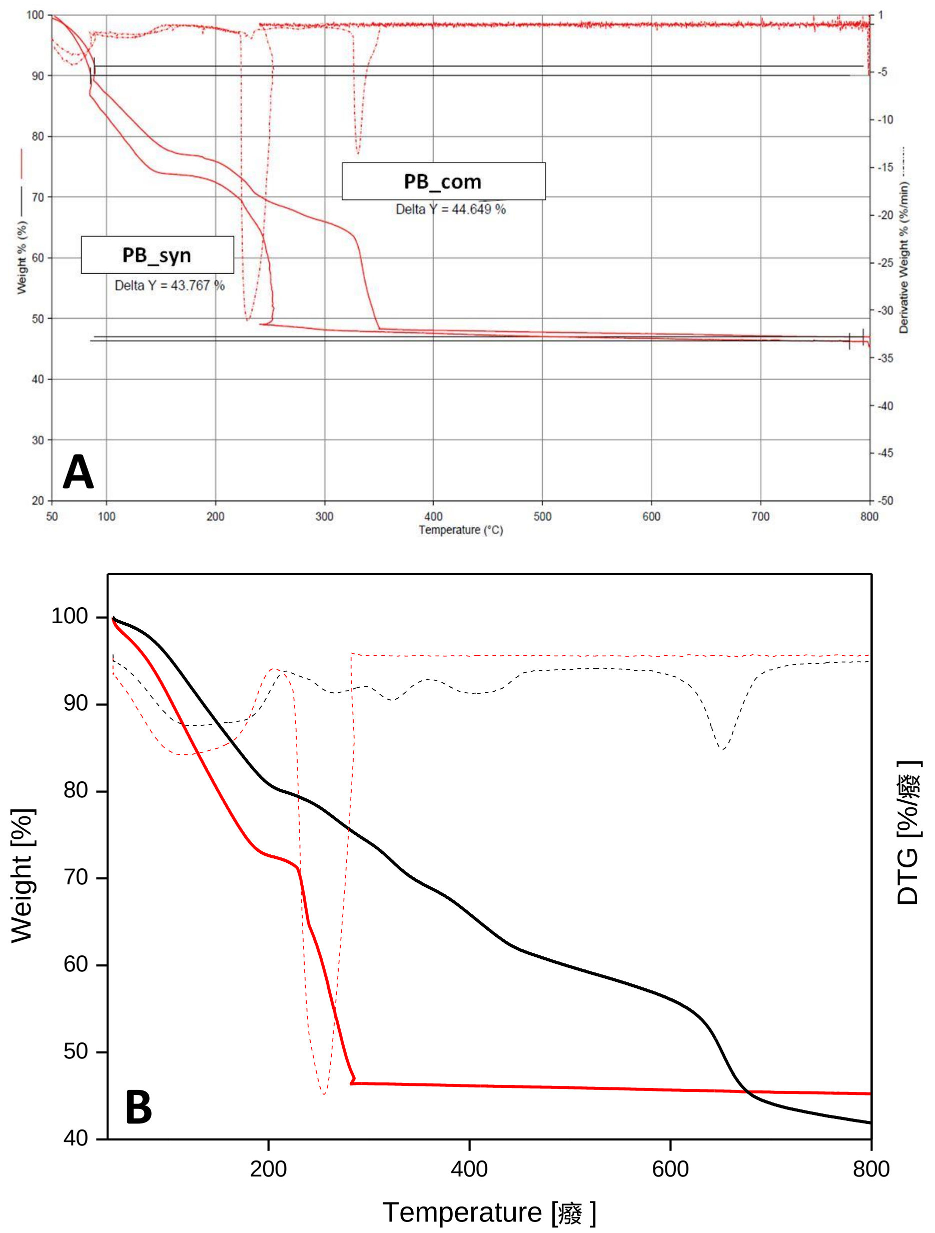

Figure S4. Frame A: TGA profile of PB_Com and PB_Syn samples. Ultrapure air flow (20 mL min^{-1}); temperature programme from 50 ℃ to 800 ℃, 5 ℃ min^{-1}; 15 mg sample. Frame: TGA profile of PB_Com (black) and PB_Syn (red) samples. First derivative profile, DTGA, as dashed curves. Ultrapure argon flow (20 mL min^{-1}); temperature programme from 50 ℃ to 850 ℃, 10 ℃ min^{-1}; 10 mg sample.

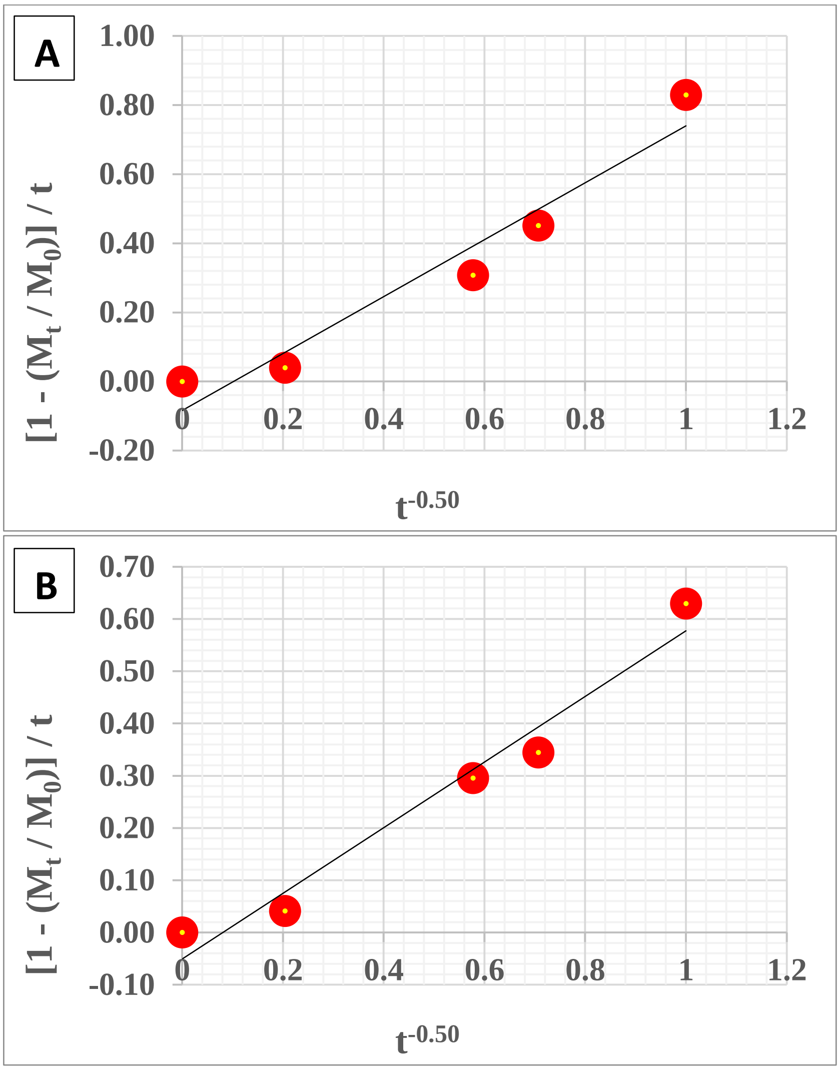

Figure S5. Plots of the kinetic parabolic model for the Cs+ uptake for PB_Syn (**A**) and PB_Com (**B**) samples, respectively.
